# Health Tract, No. 179

**Published:** 1863-12

**Authors:** 


### HEALTH TRACT, No. 179.

### STAMMERING.

It is often observed that persons in a state of intense excitement are incoherent, do not express themselves connectedly; this is simply acute stammering, resulting from a too great an amount of nervous power or influence going out in a specific direction by the mind being too intently fixed on one thing, on one idea, on one effort. The always efficient remedy is to divide the mind's attention in any way that will cause deliberation or composure. Twenty years ago it was considered a great surgical feat in the amphitheater of the University of New-York to bring in the most inveterate stutterer, and in five minutes he would go away before the wondering eyes of the students, perfectly cured, simply by having had a common knitting-needle, or its substitute, thrust through the tongue. The philosophy of this was, that unless the tongue was moved with deliberation more or less pain was excited; but the misfortune was, that as soon as the thrust was healed, the man stammered as before.

It is related in physiological works, that a laborer, the most inveterate stammerer in London, became possessed with the idea that he would make a good play-actor, and nothing that his friends could say or do could induce him to forego his resolve. The unusual circumstance gave a crowded house, and the young man went through his part without the stammer of a single syllable; because, while one effort of the mind was to remember the words and the gestures, another, a divided one, was to the utterances of his part.

My son, at the age of six, stammered inveterately. He was very impulsive and of a highly nervous temperament. Holding the views of this article, I would not allow him to be scolded or ridiculed, or have the infirmity remarked upon by any member of the family, because either of these would but increase the embarrassment or want of presence of mind; but whenever he came to me for any thing, I would say in a kindly, encouraging way: "Now, Bobby, if you will ask for it in a *slow*, plain way, you shall have it." Then, without any instruction, he would say: "Will fa-ther please give Rob-ert a piece of can-dy?" thus distinctly enunciating every syllable. I noticed at the same time, that the little fellow, at each syllable, would make a motion to strike his hand against his thigh as he stood. Here was nature's instinct coming to his aid; part of the mind, as it were, was directed to the hand keeping time to each syllable, another part to obtaining the object in view. No one ever stammers in singing, because the attention is divided between the music and the sentiment. In a few weeks little Robert ceased to stammer altogether, and has never since had the slightest trouble in that direction. Hence, the only cure for stammering is to cultivate mental deliberation in the way most easily available to each particular person.

